# Chronic Otitis Media among Patients Visiting Community-Based Static Outreach Clinics

**DOI:** 10.31729/jnma.8369

**Published:** 2023-12-31

**Authors:** Luna Mathema, Arun Adhikari, Prasanta Poudyal, Ganesh Bahadur Chalise, Preeti Chaudhary, Bijay Khatri

**Affiliations:** 1 Department of Otolaryngology & HNS, B.P. Eye Foundation, Hospital for Children, Eye, ENT, and Rehabilitation Services, Madhyapur Thimi, Bhaktapur, Nepal; 2 Academic and Research Department, B.P. Eye Foundation, Hospital for Children, Eye, ENT, and Rehabilitation Services, Madhyapur Thimi, Bhaktapur, Nepal

**Keywords:** *community health services*, *otitis media*, *patients*, *prevalence*

## Abstract

**Introduction:**

Chronic otitis media is a chronic inflammation of the middle ear and mastoid cavity, with recurrent ear discharges or otorrhoea through a tympanic perforation for the past 3 months. It is a common cause of hearing impairment, disability, and poor scholastic performance and can lead to fatal intracranial infections and acute mastoiditis. This study aimed to find out the prevalence of chronic otitis media among patients visiting community-based static outreach clinics.

**Methods:**

A descriptive cross-sectional study was conducted among patients visiting the community-based static outreach clinics from 1 January 2017 to 31 December 2019. The ethical approval was taken from the Ethical Review Board. The diagnosis of chronic otitis media was done using otoscopy. The records of patients coming to outreach clinics visiting for ear, nose and throat care were reviewed using a pre-designed study proforma. A systematic random sampling method was used. The point estimate was calculated at a 95% Confidence Interval.

**Results:**

Among 385 patients, 37 (9.61%) (6.67-12.55, 95% Confidence Interval) had chronic otitis media. The mean age of patients with chronic otitis media was 27.59±13.24 years, with 28 (75.67%) patients aged between 18-60 years. Among them, 30 (81.08%) had unilateral and 34 (91.89%) had a mucosal type.

**Conclusions:**

The prevalence of chronic otitis media was lower than in other studies done in similar settings.

## INTRODUCTION

Chronic otitis media (COM) is a common cause of hearing impairment, disability, and poor scholastic performance.^1^ If left untreated, it can lead to fatal intracranial infections and acute mastoiditis, especially in developing countries.^1^ Illiteracy, frequent upper respiratory tract infections, poor socioeconomic conditions, poor hygiene, and poor nutrition are often associated with the development of COM.^2,3^

The World Health Organization (WHO) estimates that the prevalence of COM varies from region to region from as low as 0.2% to as high as 10%, and 7.8% in Southeast Asia.^4^ A prevalence of 4% or greater indicates a public health problem that needs urgent attention.^5^ Early detection and initiation of treatment are crucial to have better outcomes and prevent complications. However, only a few studies have been published about the burden of COM in Nepal.

This study aimed to find out the prevalence of COM among patients visiting community-based static outreach clinics.

## METHODS

This descriptive cross-sectional study was conducted at five static outreach clinics (SORC) inside the Kathmandu Valley under the Hospital for Children, Eye, ENT, and Rehabilitation Services (CHEERS), Madhyapur Thimi, Bhaktapur, Nepal. The five SORCs included Changunarayan Municipality Hospital, Siddhi Memorial Hospital and Bode Urban Clinic of Bhaktapur district, Bajrabarahi Community Hospital of Lalitpur district and Manmohan Community Hospital of Kathmandu district. The ethical approval was taken from the Ethical Review Board of Nepal Health Research Council (Registration number: 12/2021 P). The records of patients visiting the ENT clinics between 1 January 2017 to 31 December 2019 were reviewed and recorded from 1 March 2021 to 31 May 2021. The record review permission was taken from CHEERS. Only those patients whose records were available and complete in the SORC register records were selected for the study. Data that were not legible to read were excluded from our study. A systematic random sampling method was used. The sample size was estimated using the following formula:


$$\begin{array}{ll} \text{n} & ={\text{Z}}^{2}\times \frac{\text{p}\times \text{q}}{{\text{e}}^{\text{2}}}\\ & =1.96^{2}\times \frac{0.50\times 0.50}{0.05^{2}}\\ & =385 \end{array}$$

Where,

n = minimum required sample size
Z = 1.96 at a 95% Confidence Interval (CI)
p = prevalence taken as 50% for maximum sample size calculation
q = 1-p
e = margin of error, 5%

The calculated minimum required sample size was 385.

The daily record registers from outreach clinics were reviewed and the data were entered in chronological order as per the registration number. The record review showed that 4,204 patients had visited the clinic during the study period. The sampling interval "k" was calculated using the following formula for systematic random sampling:

k= N/n

 = 4204/385

 = 10.92 ≅ 11

Where,

k = sampling interval
N = estimated total population during the study period
n = minimum required sample size

Using a computer-generated random number, '9' was selected as the starting point, and then every 11^th^ item was selected till the minimum sample size was reached.

COM was defined as chronic inflammation of the middle ear and mastoid cavity, with recurrent ear discharges or otorrhoea through a tympanic perforation for the past 3 months. The patients at SORCs were diagnosed using otoscopy and treated by community ear health workers (CEHWs). The CEHWs are trained extensively for 3 months to deliver primary ear care services in the community, including identifying and treating common ear conditions, referral, and ear health promotion. The record review proforma was developed from the literature review and the advice of fellow ENT-HNS Surgeons, Consultant Audiologists, and Public Health experts.

The collected data were entered in Microsoft Excel 2019 and analyzed using IBM SPSS Statistics version 26.0. The point estimate was calculated at 95% CI.

## RESULTS

Among 385 patients, 37 (9.61%) (6.67-12.55, 95% CI) had COM. Among the patients with COM, 34 (91.89%) had a mucosal type, and 3 (8.11%) had a squamous type. A total of 7 (18.92%) cases were bilateral, and the remaining 30 (81.08%) were unilateral (Table 1). All squamous COMs were unilateral.

**Table 1:** Distribution of COM according to types and ear affected (n= 37).

Characteristics	n (%)
**Type**	
COM mucosal	34 (91.89)
COM squamous	3 (8.11)
**Ear affected**	
Both ears	7 (18.92)
One ear	30 (81.08)

The mean age of patients with COM was 27.59±13.24 years, and that of males and females were 28.85±11.97 and 24.40±16.42, respectively. Among them, 28 (75.67%) patients with COM were adults aged 18-60 years old, and 10 (27.03%) were females (Table 2).

**Table 2:** Baseline characteristics of patients with COM (n= 37).

Characteristics	n (%)
**Age (years)**	
6-17	9 (24.33)
18-60	28 (75.67)
**Gender**	
Male	27 (72.97)
Female	10 (27.03)

## DISCUSSION

The prevalence of COM in our study was 9.61%, which is lower than a study conducted in camp settings in the plain Terai belt of Nepal, where 14.21% had COM.^6^ A hospital-based study in eastern Nepal also had a higher prevalence, where 21.36% of patients coming to ENT OPD had COM.^7^ Most people in the Terai practice swimming and bathing in ponds and rivers without protection, which might relate to the higher prevalence in Terai as shown in studies from Bangladesh.^8,9^ In addition, the higher prevalence in the hospital of eastern Nepal might be attributed to the fact that the hospital is a tertiary referral centre. Nevertheless, the burden indicates that COM is a public health problem and needs urgent attention in Nepal.

In our study, 81.08% of cases were unilateral COM. A study in a tertiary hospital in Tanzania also showed unilateral involvement (97.5%) was commoner than bilaterality (2.5%).^10^ Topical quinolones are effective in resolving otorrhoea and eliminating the microorganism and are the treatment of choice.^11^ If not treated, COM can lead to hearing loss.^12^ Similarly, in our study, mucosal COM was more common (91.89%). In a study from ear camps of Sunsari and Morang districts of Nepal, 95.6% had COM mucosal type.^6^ In another study in a hospital in Eastern Nepal, 98.87% of patients with COM belonged to the mucosal group.^7^ In another study among patients attending ENT OPD in Dhulikhel Hospital of Nepal, the mucosal type was also the predominant type of COM with 60% of total cases.^13^ The mucosal COM, often called a safe type, can be treated with aural toileting and medicine or surgery. However, if not treated on time, it can lead to sensorineural hearing loss, and patients may need rehabilitation with hearing aids.^14^

In our study, more than three-fourths (75.67%) of patients with COM were adults. The camp settings study in the eastern Terai region also showed that 61.9% of patients with COM were from a similar age group.^6^ The disease usually begins in childhood,^4^ but can spill over into adulthood unless the affected drum is repaired surgically.^14^ The lower proportion of children in the studies in Nepal might also be due to fewer from those age groups coming to the SORC and camp settings. So, school ear health care programs should be conducted in developing countries like Nepal to reach the children population.

Among patients with COM, our study showed that the condition was higher in males (72.97%) than females (27.03%). The eastern Nepal hospital-based study also shows the same, with 1,776 male patients and 1,589 female patients having COM.^7^ The Dhulikhel hospital study also showed that males were more affected than females.^13^ A male predominance was also found at a University Clinic in Kinshasa, Tanzania.^10^ Clinically, respiratory tract infection is often associated with COM,^2^ and a review has also shown that males are more susceptible and develop such infection more frequently than females.^15^ Besides, the male predominance in all these studies can be due to women facing multiple barriers to accessing healthcare, such as limited availability of healthcare facilities, financial constraints, lack of transportation, or socio-cultural norms restricting women's mobility. Community outreach and health programs targeting disadvantaged populations, including women, need to be conducted to address health disparities and improve overall community well-being.

Ear problems are neglected in Nepal's health system. The specialized human resources for ear care are centralized in metro and urban areas, with negligible services in rural areas or even on the outskirts of urban areas. Besides, the knowledge, awareness, and health-seeking behaviour among community people about COM is not satisfactory in Nepal. One of the ways to address ear health issues is by developing a cadre for primary ear care like CEHWs, and conducting different medical camps or from SORC. COM typically produces mild to moderate conductive hearing loss. It is estimated that COM may contribute more than half to the global burden of hearing impairment, and eliminating it can potentially reduce the global burden by four-fifths.^4^ Nevertheless, health promotion measures like breastfeeding, immunization, adequate nutrition, personal hygiene, improved housing, reduced overcrowding, and adequate access to clean water can reduce its prevalence.^5^

The major limitation of our study is that the study was conducted in once-a-week SORCs in the outskirts of Kathmandu Valley and may not be generalizable to other institutional settings.

## CONCLUSIONS

The prevalence of COM was lower than in other studies done in similar settings.
